# Treatment of pneumothorax and bronchopleural fistula by extracorporeal membrane oxygenation in a neonate: a case report

**DOI:** 10.3389/fped.2024.1466852

**Published:** 2024-12-23

**Authors:** Taining Zhang, Jianxiu Wang, Bingyin Zhou, Bin Yan, Xilong Chen, Yanxia Wang, Weikai Wang

**Affiliations:** Department of Pediatric Intensive Care Unit, Gansu Provincial Maternity and Child Care Hospital, Lanzhou, Gansu, China

**Keywords:** extracorporeal membrane oxygenation (ECMO), neonate, pneumothorax, bronchopleural fistula (BPF), case report

## Abstract

Intractable pneumothorax secondary to bronchopulmonary fistula is a rare complication in neonates. We present the first report of a newborn with spontaneous pneumothorax and bronchopleural fistula treated with extracorporeal membrane oxygenation (ECMO). Positive pressure mechanical ventilation resulted in persistent air leakage from the bronchopleural fistula. When ECMO was initiated, a “total lung rest” ventilation strategy was used to facilitate the healing of bronchopleural fistula and the absorption of the pneumothorax. ECMO is an effective supportive therapy for neonate pneumothorax and bronchopulmonary fistula when conventional ventilation management fails.

## Introduction

Pneumothorax, a collection of free air in the pleural space, is a common condition in neonates, with an incidence of 0.8–1.9 per 1,000 live births (1). Treatment strategies for neonates' pneumothorax are based on their clinical status and vary from conservative management to drainage with needle aspiration or intercostal catheter insertion (2). When the symptoms are not relieved by multiple well-placed drains, a bronchopleural fistula (BPF) should be suspected. BPF is defined as a sinus tract between the pleural cavity and bronchus with persistent air leak. In mechanically ventilated patients, air leaks persisting beyond 24 h may suggest the presence of BPF (3).

Intractable pneumothorax secondary to BPF is a rare complication of the neonate respiratory system, with a high mortality rate (4). Suggested first-line management includes chest tube insertion, patient positioning, and ventilation strategies such as high-frequency ventilation (4). Ventilation treatment to minimize air leaks and optimize gas exchange requires a full understanding of the physiologic mechanisms of BPF airflow. Laminar flow through a BPF is an interaction of visceral pleural defect size, local lung compliance, airway pressure, relative airway resistance, and transpulmonary pressure (5). However, when ventilation is ineffective, other more advanced options should be considered.

Extracorporeal membrane oxygenation (ECMO) is a temporary artificial extracorporeal treatment for respiratory and cardiac failure when conventional therapies have failed (6). It is a life-saving therapy and plays an essential role in neonatal critical illness (7). ECMO was first used to support neonatal respiratory failure in 1975 (8), with an overall survival rate of 87% in 33,400 neonates worldwide, as reported by the extracorporeal life support organization (ELSO) (9). During ECMO, a lung-protective ventilation strategy is usually used to reduce ventilator-related lung injury to ensure the rest and recovery of the lungs. In patients with severe air leakage, ventilator parameters are lowered to allow the lungs to rest completely, and the alveoli may collapse either entirely or partially. As the patient stabilizes and the lung condition improves, the collapsed lung tissue needs to be recruited to improve oxygenation (10).

We present a case of a newborn with spontaneous pneumothorax complicated with BPF treated with ECMO. We sought to determine if venoarterial ECMO (VA-ECMO) could provide a bridge to lung-protective ventilation and etiologic treatment and allow bronchial healing in neonates with pneumothorax and BPF.

## Case report

An 11-h-old male newborn was transferred to our tertiary hospital from another medical institution due to respiratory distress on July 20, 2023. The patient was the second-born child with a gestational age of 38 weeks and 4 days. He was born by cesarean section with a birth weight of 2,850 g. His 1- and 5-min Apgar scores after birth were 8 and 7, respectively.

**Figure 1:**
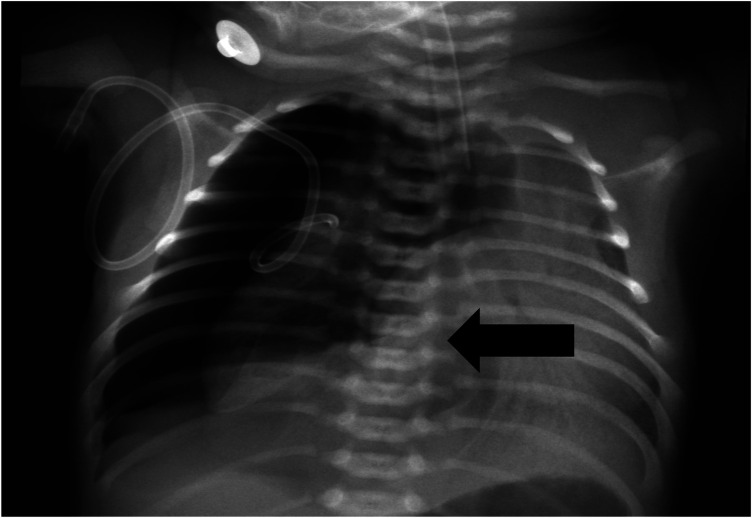
Chest x-ray showing right-sided pneumothorax with mediastinal shift.

When the patient was transferred to our hospital, he was experiencing significant respiratory distress. An urgent chest x-ray revealed right-sided pneumothorax along with mediastinal and tracheal shift to the opposite side, confirming tension pneumothorax (Figure 1). The newborn was diagnosed with meconium aspiration syndrome (MAS), complicated by BPF. After several days of positive pressure ventilation, the child's pulmonary findings and oxygenation did not demonstrate significant improvement. Therefore, we concluded that the primary factor contributing to this situation was the alteration in lung pressure dynamics due to positive pressure ventilation, which ultimately led to the persistent presence of BPF.

Following a comprehensive discussion with the surgeon, closed chest drainage was implemented. He was started on high-frequency oscillatory ventilation (HFOV) with a chest tube of 6.5 Fr inserted. The system utilized a 6.5 F inner diameter, and the negative pressure generated by the closed chest drainage under a 1-m gravity drop condition proved adequate for effective drainage. The primary setup of the HFOV was as follows: MAP: 10 cmH2O, amplitude: 20 cmH2O, frequency 9 Hz, FIO2: 100%. The patient had dyspnea, and the blood gas analysis was as follows: PH 7.25, PCO2 52 mmHg, PO2 39 mmHg, HCO3− 20.6 mmol/L, BE −4.8 mmol/L, Lac 1.5 mmol/L. The HFOV setting was adjusted as follows: MAP: 11 cmH2O, amplitude: 22 cmH2O, frequency 8 Hz, FIO2: 100%. The repeated blood gas analysis was as follows: PH 7.40, PCO2 38 mmHg, PO2 59 mmHg, HCO3− 24 mmol/L, BE −1.0 mmol/L, Lac 1.4 mmol/L. The patient's dyspnea improved, and the HFOV settings were adjusted as follows: MAP: 10 cmH2O, amplitude: 20 cmH2O, frequency 8 Hz, FIO2: 70%–85%. However, the patient's dyspnea worsened again, and the repeated blood gas analysis was as follows: PH 7.23, PCO2 57 mmHg, PO2 51 mmHg, HCO3− 21.2 mmol/L, BE −4.3 mmol/L, Lac 0.6 mmol/L. The HFOV setting was adjusted as follows: MAP: 10 cmH2O, Amplitude: 20 cmH2O, Frequency 9 Hz, FIO2: 100%. Blood gas analysis was as follows: PH 7.07, PCO2 59 mmHg, PO2 72 mmHg, HCO3− 14.3 mmol/L, BE −13.6 mmol/L, Lac 4.2 mmol/L. We immediately conducted volume expansion via saline, thoracentesis, and closed drainage and rechecked the blood gas analysis after 1 h as follows: PH 7.19, PCO2 36 mmHg, PO2 53 mmHg, HCO3− 14.2 mmol/L, BE −13.5 mmol/L, Lac 2.7 mmol/L.

Three days after the HFOV mode of ventilator-assisted respiratory therapy, the patient frequently had decreased percutaneous oxygen saturation (SPO2) and dyspnea. Multiple changes and adjustments were made to his chest tube position. This further compromised lung ventilation as minimal changes to drain position may lead to significant respiratory deterioration and aggravated pneumothorax. Oxygenation and CO2 exchange remained challenging, and the patient's condition was in line with the indications of ECMO for neonatal respiratory failure (11). Therefore, ventilator parameters were down-regulated, and ECMO treatment was started.

**Figure 2:**
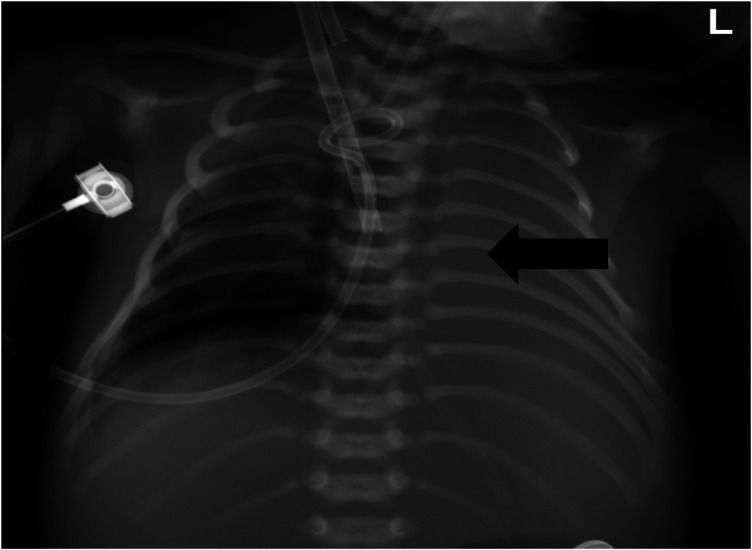
Chest x-ray showed that the right pneumothorax was significantly reduced, and the left lung was not fully expanded.

**Figure 3:**
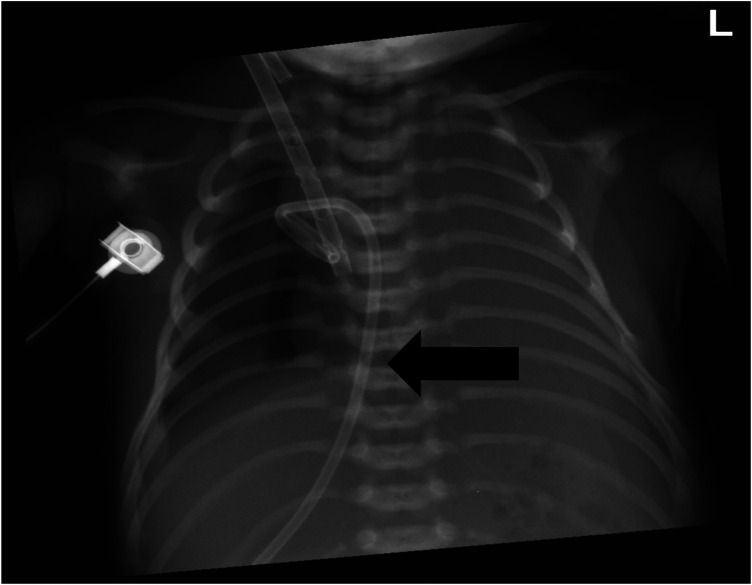
Chest x-ray showing partial improvement of right pneumothorax.

**Figure 4:**
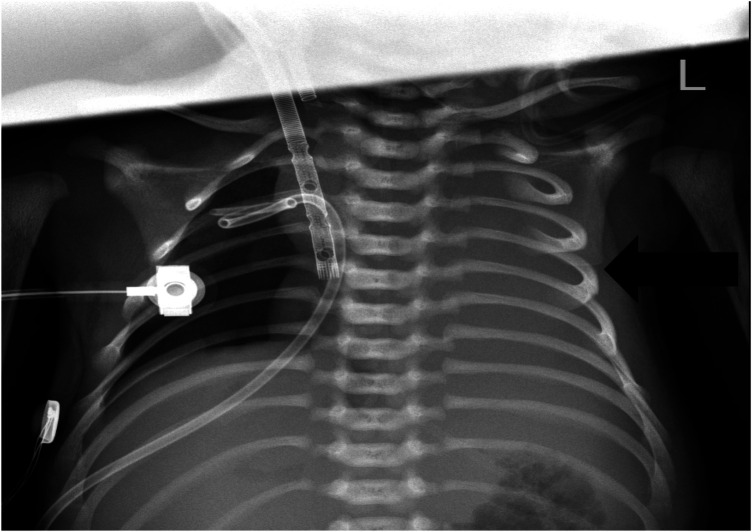
Chest x-ray showing aggravated pneumothorax in the right lung and exudative pleural effusion in the left lung.

VA-ECMO support was started with the following initial settings: blood flow: 0.6 L/min; sweep gas flow: 0.275 L/min; and oxygenator FiO2: 0.7. Thereafter, the mechanical ventilation settings were gradually titrated to “lung rest” parameters: respiratory rate (RR): 20/min; PEEP: 4 cmH2O; peak inspiratory pressure (PIP): 20 cmH2O; inspiratory time (iT): 0.6 s; and FiO2: 0.3. The patient's PaO2 increased to 82 mmHg, and PaCO2 decreased to 34 mmHg. Intermittent prone position ventilation was performed every 12 h to reduce lung inflammation from day 5. On the third day after ECMO, the chest x-ray showed that pneumothorax in the right lung was significantly reduced, and the left lung was not fully expanded (Figure 2). We gradually increased PEEP at 1 cmH2O/h for lung recruitment. The chest tube was fixed smoothly, without falling off or shifting, and a large number of bubbles appeared in the water-sealed bottle. On the fourth day after ECMO, the chest x-ray only showed partial improvement in pneumothorax (Figure 3), and we continued increasing PEEP. On the fifth day after ECMO, the patient's dyspnea was aggravated with increased PEEP, and the chest x-ray showed aggravation of the right pneumothorax (Figure 4). We had increased PEEP for a total of four times, finally reaching 8 cmH2O. Based on the clinical and radiological findings, a BPF was suspected. Continuous positive pressure ventilation resulted in the BPF valve remaining open, which constituted the primary cause of the persistent air leak.

**Figure 5:**
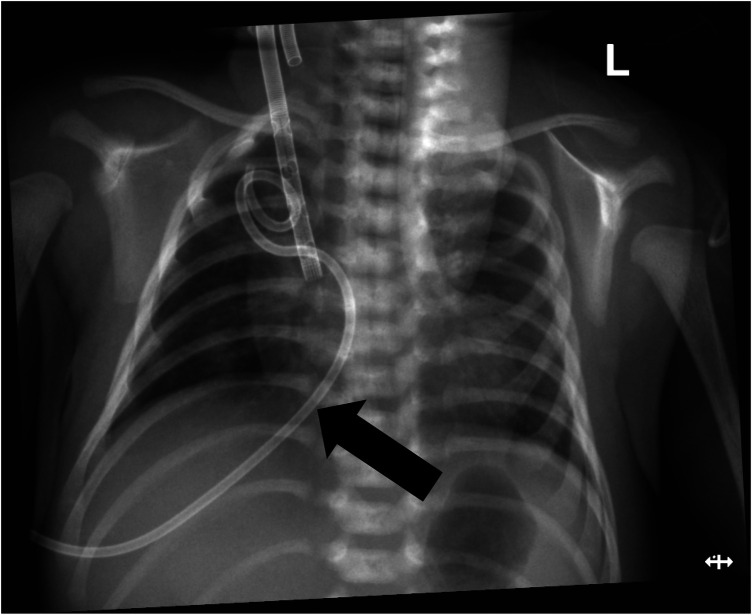
Chest x-ray showing resolved pneumothorax.

**Figure 6:**
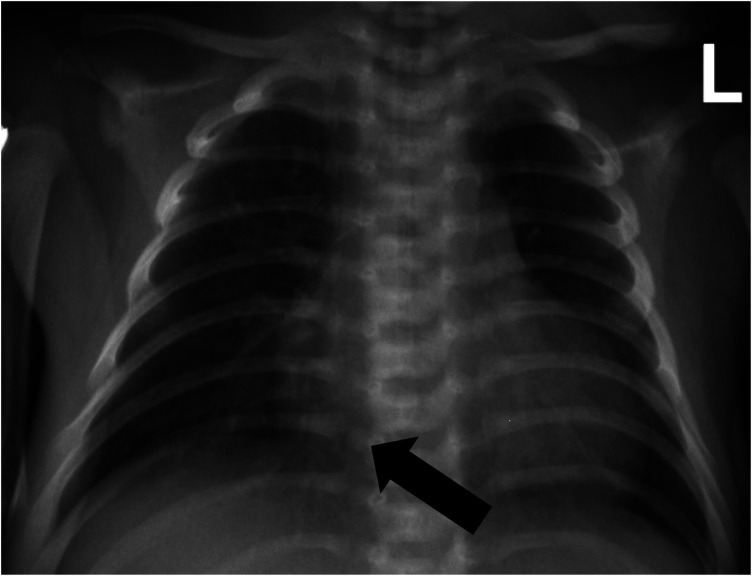
Chest x-ray showing improved exudative lesions in both lungs.

On day 8, to allow a good rest of the lungs, we removed the tracheal intubation and stopped the ventilator-assisted ventilation after expert consultation and a literature review (12–14). The patient was not on a nasal cannula for oxygen since ECMO had already provided sufficient oxygen for the child. ECMO and supportive treatment were continued. On day 10, chest x-ray showed no pneumothorax (Figure 5), and the patient was started on PSV-SIMV mode ventilator-assisted ventilation again. However, the patient had dyspnea with aggravated pneumothorax after the ventilation. Considering the BPF was not healed, the tracheal intubation was pulled out again. Two days later, the chest tube showed no gas outflow, and the chest x-ray showed no obvious pneumothorax. Ventilator-assisted ventilation was started again. The patient's blood gas analysis and SpO2 remained normal. No pneumothorax was found in chest x-ray at 12 h and 24 h after intubation, suggesting that BPF healed by itself. On day 14, the patient was successfully disconnected from ECMO. Ventilation was continued, and anti-infection and other symptomatic treatments were given. On day 17, he gradually improved and was extubated to non-invasive respiratory support. High PEEP was initially provided to prevent airway collapse (Figure 6). On day 23, the patient's condition improved, and he was discharged.

## Discussion

To the best of our knowledge, we are reporting the first case of successful management of neonate pneumothorax and BPT by awake ECMO. In patients with pneumothorax and BPF that are refractory to ventilation treatment, veno-venous-extracorporeal membrane oxygenation (VV-ECMO) promotes gas exchange while permitting ultralung protective ventilation. ECMO allows for BPF healing and fistula closure, which may provide a bridge to a definitive intervention. Case reports have illustrated the success of ECMO in BPF among various patients (14–18). In some cases, an early tracheostomy is performed after ECMO initiation, and the patient is extubated to quickly wean from sedation and mechanical ventilation. This strategy minimizes or eliminates the need for positive pressure ventilation.

The key to the patient's recovery in this case was the combination of negative-pressure spontaneous breathing, allowing total atelectasis to seal the BPF while ECMO provided oxygenation and ventilation. Both positive and negative pressure ventilation techniques have been extensively studied and compared across various populations, including neonates (19–21). A recent literature review showed that the majority of studies demonstrated the advantages of positive pressure extubation techniques in maintaining stable vital signs, preventing complications during the peri-extubation period, and improving overall survival (22). Katira and Cereda (23) suggests that negative pressure is a positive way to breathe. Vincenzi (24) introduced a new mode of mechanical ventilation by combining positive and negative synchronized ventilation. Our study makes a unique contribution to the literature by showing the benefits of combining negative-pressure breathing and positive-pressure support.

Awake ECMO has attracted increasing interest in clinical practice recently, but its application in neonates is relatively rare. Costa et al. (14) reported eight neonates who were electively extubated while on ECMO, among whom five had expedited resolution of pneumothorax and three had successful lung recruitment, enabling further decannulation from ECMO. Our findings are consistent with Costa et al.'s (14) study and suggest that awake neonatal ECMO has advantages over traditional management in terms of safety and effectiveness in certain clinical conditions, especially for patients with air leaks. In addition, our case illustrates that ECMO alone is often not enough in rest ventilator settings. We observed the patient's resolution under spontaneous breathing, followed by recurrence under positive pressure ventilation, and then resolution again under spontaneous breathing. These alternations suggest that a certain degree of volume loss is sometimes required to achieve the resolution of BPF, which can be safely and uniquely supported by the concomitant use of ECMO.

Venoarterial ECMO (VA-ECMO) has also been used in postoperative patients unable to undergo primary BPF closure. In a case series of five patients with massive air leaks (>5 ml/kg), VA-ECMO led to BPF closure in three of five patients (25). The major advantage of VA-ECMO is the ability to stabilize patients with respiratory or cardiac failure, thus making it the most appropriate technique in this life-threatening condition. In patients with pneumothorax and BPF with persistent air leaks, VA-ECMO allows the use of lung-protective ventilation, followed by ECMO weaning, extubation, and BPF healing.

However, it should be noted that VA-ECMO is a high-risk strategy for isolated air leaks, as empirical evidence has consistently shown that VA-ECMO is associated with a higher risk of complications and even mortality than VV-ECMO (26–28). In this study, we chose VA-ECMO as our first option because there are currently no appropriate devices available in China for neonates weighing less than 15 kg to establish an extracorporeal circulation pathway via a veno-venous double-lumen catheter for VV-ECMO support. In the US, 13 Fr Crescent RA cannula has well-established effectiveness for VV-ECMO support in neonates, but it is not yet available in Asia. Consequently, we are compelled to utilize VA-ECMO for the management of severe pulmonary conditions in this case.

In this study, initiation of VA-ECMO was set at a high level of 210 ml/kg/min, which is typical for neonates to maintain a high blood flow, generally at 150 ml−200 ml/kg/min. The reason is that neonates have relatively low antithrombin III activity, leading to few substrates that bind to the heparin anticoagulation used in the ECMO transfer process. Therefore, we maintained a relatively high flow rate during clinical treatment to ensure the smooth operation of ECMO and prevent potential pulmonary embolism caused by thrombus.

Additionally, we maintained a relatively low PEEP of 4 cmH2O and a standard rest PIP of 20 cmH2O in the “lung rest” ventilator settings. In the context of ventilator parameter settings during ECMO assistance, it is essential to maintain adequate lung recruitment and blood flow through appropriate PEEP support. However, with the growing emphasis on awake ECMO, positive pressure ventilation presents several disadvantages. The alternative role of ECMO in supporting lung function can further mitigate the risks associated with barotrauma and capacity injury. For patients who have already experienced significant air leakage, ECMO sufficiently meets the body's oxygen demands. Awake ECMO facilitates autonomous breathing while reducing respiratory workload, thereby minimizing complications related to positive pressure ventilation. Consequently, this forms our primary physiological rationale for extubation.

In this study, PEEP was increased four times, which may partially explain the aggravation of pneumothorax during lung recruitment. Reducing PEEP can improve pneumothorax in the right lung but will also worsen the atelectasis in the left lung, causing excessive alveolar collapse and even permanent atelectasis. It is a dilemma to adjust the ventilator to reduce the pneumothorax of the right lung and recruit the left lung at the same time. Therefore, we can only increase PEEP within an appropriate range to achieve the best treatment effect. Later, when we lowered PEEP, the pneumothorax improved. So, we finally considered the “whole lung rest” strategy to promote the self-healing of bronchopleural leakage.

Management of pneumothorax and BPF requires appropriate mechanical ventilation with necessary adjustments that best fit the changing conditions of the patient, such as the combination of negative-pressure breathing and positive-pressure support. Strategies include decreasing the following indicators to decrease the airflow across the BPF: peak inspiratory pressure, tidal volumes, positive end-expiratory pressure, inspiratory time, respiratory rate, and negative intrapleural pressure. Our findings suggest that when traditional therapies such as HFOV fail, ECMO (including awake ECMO) should be considered as it has the potential to resolve air leaks while avoiding the hemorrhagic risks associated with invasive drainage, which can improve clinical outcomes and survival. At the same time, caution should be given to the potential risk related to ECMO, particularly VA-ECMO.

## Data Availability

The original contributions presented in the study are included in the article/Supplementary Material, further inquiries can be directed to the corresponding author.
