# Supplementary figures and images for: Treatment of pneumothorax and bronchopleural fistula by extracorporeal membrane oxygenation in a neonate: a case report
Source: Front Pediatr. 2024 Dec 23;12:1466852. doi: 10.3389/fped.2024.1466852 (PMC11701141; doi:10.3389/fped.2024.1466852)

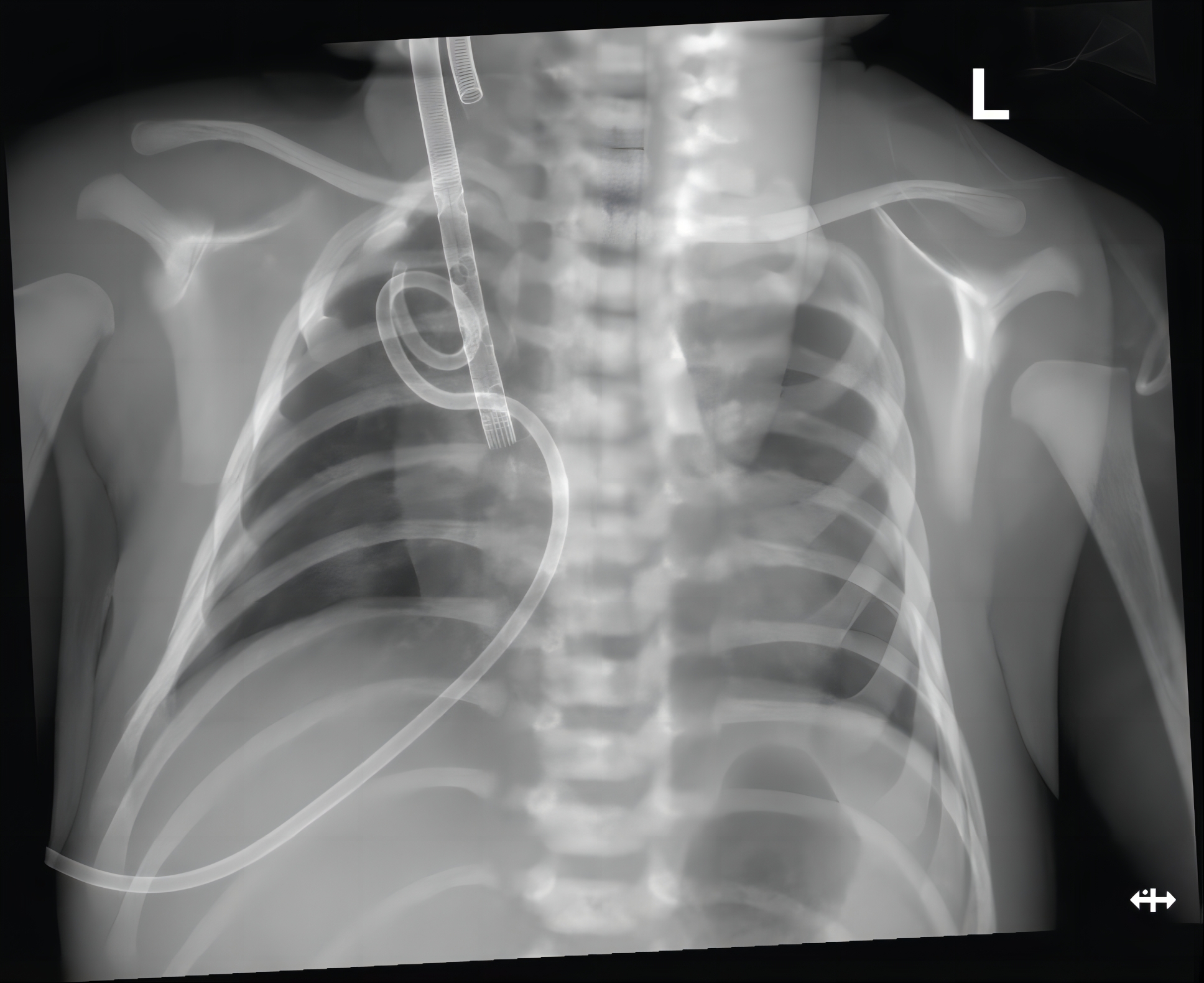

Supplement: Supplementary file 1 [file Image1.jpeg]

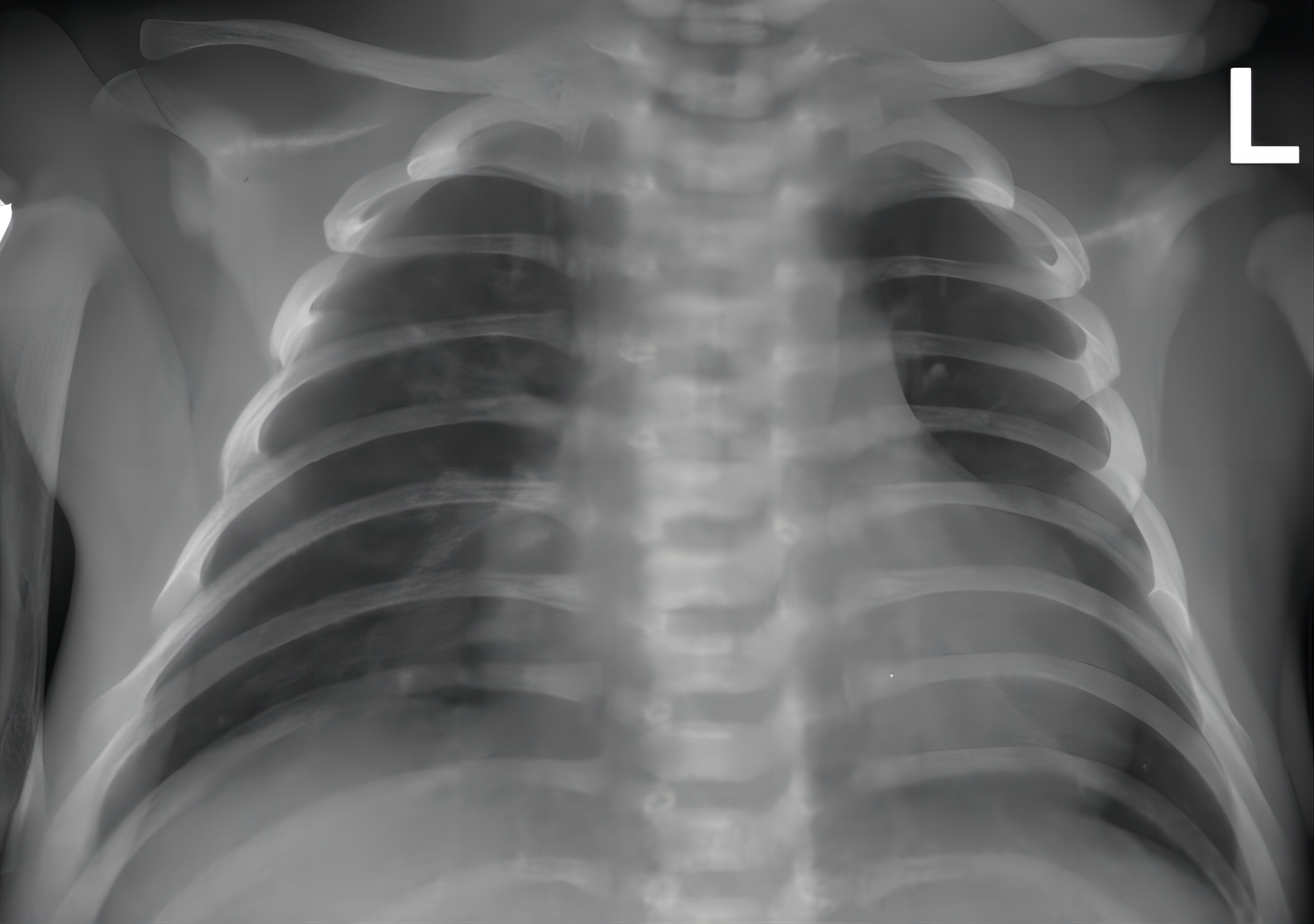

Supplement: Supplementary file 2 [file Image2.jpeg]
